# The effects of 4-Phenylbutyric acid on ER stress during mouse tooth development

**DOI:** 10.3389/fphys.2022.1079355

**Published:** 2023-01-04

**Authors:** Eui-Seon Lee, Yam Prasad Aryal, Tae-Young Kim, Elina Pokharel, Ji-Youn Kim, Hitoshi Yamamoto, Chang-Hyeon An, Seo-Young An, Jae-Kwang Jung, Youngkyun Lee, Jung-Hong Ha, Wern-Joo Sohn, Jae-Young Kim

**Affiliations:** ^1^ Department of Biochemistry, Daegu, South Korea; ^2^ Department of Dental Hygiene, Gachon University, Incheon, Korea; ^3^ Department of Histology and Developmental Biology, Tokyo Dental College, Tokyo, Japan; ^4^ Department of Oral and Maxillofacial Radiology, Daegu, South Korea; ^5^ Department of Oral Medicine, Daegu, South Korea; ^6^ Department of Conservative Dentistry, School of Dentistry, IHBR, Kyungpook National University, Daegu, Korea; ^7^ Department of K-Beauty Business, College of Cosmetics and Pharmaceuticals, Daegu Haany University, Gyeongsan, Korea

**Keywords:** chemical chaperones, dentinogenesis, morphogenesis, secretion, signaling

## Abstract

**Introduction:** During tooth development, proper protein folding and trafficking are significant processes as newly synthesized proteins proceed to form designated tissues. Endoplasmic reticulum (ER) stress occurs inevitably in tooth development as unfolded and misfolded proteins accumulate in ER. 4-Phenylbutyric acid (4PBA) is a FDA approved drug and known as a chemical chaperone which alleviates the ER stress. Recently, several studies showed that 4PBA performs therapeutic effects in some genetic diseases due to misfolding of proteins, metabolic related-diseases and apoptosis due to ER stress. However, the roles of 4PBA during odontogenesis are not elucidated. This study revealed the effects of 4PBA during molar development in mice.

**Methods:** We employed in vitro organ cultivation and renal transplantation methods which would mimic the permanent tooth development in an infant period of human. The *in vitro* cultivated tooth germs and renal calcified teeth were examined by histology and immunohistochemical analysis.

**Results and Discussion:** Our results revealed that treatment of 4PBA altered expression patterns of enamel knot related signaling molecules, and consequently affected cellular secretion and patterned formation of dental hard tissues including dentin and enamel during tooth morphogenesis. The alteration of ER stress by 4PBA treatment during organogenesis would suggest that proper ER stress is important for pattern formation during tooth development and morphogenesis, and 4PBA as a chemical chaperone would be one of the candidate molecules for dental and hard tissue regeneration.

## Introduction

Tooth development starts from thickening of oral epithelium followed by the migration of neural crest-derived mesenchyme below the epithelium (Jussila and Thesleff, 2012; Balic, 2018). Interactions between epithelium and the underlying mesenchyme play important roles for development of tooth from bud, cap, bell to secretory stages (Santosh and Jones, 2014; Balic, 2018). At cap stage, enamel knot (EK), a non-proliferating signaling center of tooth development closely linked with epithelial-mesenchymal interactions, expresses important signaling molecules including autocrine and paracrine factors, and transcription factors for proper tooth morphogenesis (Yu and Klein, 2020; Abramyan et al., 2021). At the secretory stage, inner enamel epithelium (IEE) begins differentiate into ameloblasts and the underlying mesenchymal dental papilla (DP) differentiate into odontoblasts for the secretion of enamel and dentin respectively (Otsu et al., 2011; Kawashima and Okiji, 2016). However, when there is disturbance in the secretion of extracellular matrix (ECM) proteins, it may lead to Amelogenesis Imperfecta (AI) or Dentinogenesis Imperfecta (DI) (Smith et al., 2017). It is suggested that AI would be one of the results of ER stress which arises when secreting cells such as ameloblasts have inadvertent stress (Brookes et al., 2014, 2017; Aryal et al., 2019).

The regulation of ER stress is important for proper modulation of cell signaling and secretion during tooth development (Kim J. W. et al., 2014; Brookes et al., 2014, 2017; Aryal et al., 2020). ER stress occurs when the capacity of protein-folding of ER is exceeded (Tabas and Ron, 2011). As ER stress occurs, it induces the phenomenon called unfolded protein response (UPR) in order to maintain protein homeostasis through collection of intracellular signal transduction reactions (Sano and Reed, 2013). During ER stress regulation, the binding immunoglobulin protein (BiP) binds the unfolded proteins in the ER lumen for proper folding of unfolded and misfolded proteins (Pobre et al., 2019). ER stress triggers accumulation of unfolded proteins which leads to nutrient deprivation, hypoxia and point mutation (Zhang et al., 2010; Oakes and Papa, 2015). Recent studies in ER stress revealed that ER stress modulation plays an important role in hard tissue formation, especially dentin formation (Brookes et al., 2014, 2017; Aryal et al., 2019).

The chemical chaperones correct the folding and assemblance of proteins, thus controlling protein trafficking by preventing their degradation or aggregation for proper protein function to alleviate ER stress (Kim et al., 2013; Cortez and Sim 2014). Because of their proteostatic function, it draws increasing attention for many therapeutic agents. Phenylbutric acid (4PBA) is a chemical chaperone in the ER which enhances the capacity of protein folding and also facilitates the proper folding of mutant proteins (Iannitti and Palmieri, 2011; Kim et al., 2013). The clinical use of 4PBA as an ammonia scavenger has been approved (Uppala et al., 2017). In addition, some studies proved the efficacy of 4PBA in metabolic diseases such as obesity and diabetes and in inflammatory diseases (Luo et al., 2010; Kim et al., 2013; Chen et al., 2016; Wu et al., 2020). However, the effects of 4PBA during dodntogenesis has not yet elucidated. Based on our previous study, which revealed the expression patterns of ER stress related signaling molecules during various stages of tooth development (Aryal et al., 2020), thus, we designed to examine the role of 4PBA in tooth development using *in vitro* organ cultivation system.

## Materials and methods

The animal experiments performed in this study were ethically approved (KNU 2020-0107). We followed the guidelines from Intramural Animal Use and Care Committee, Kyungpook National University, School of Dentistry.

## Animals

The healthy time-mated pregnant ICR mice at embryonic stage 14 (E14) were obtained for embryo collection. Similarly, adult ICR 7-week old male mice were used for renal transplantation experiment. Mice were housed in optimum conditions, including room temperature (22°C ± 2°C), 55 ± 5% humidity and artificial illumination with lights on from 05:00 to 17:00 h, with access to food and water *ad libitum*.

### *In vitro* organ cultivation and drug treatment

Tooth germs at E14 stage were micro-dissected in PBS and then cultivated with either 100 μM 4PBA (Sigma Aldrich, St. Louis, Missouri, United States) or 0.07% DMSO with DMEM (HyClone, Logan, UT, United States; cat. no.-SH30243.01) and 10% fetal bovine serum (Hyclone, Logan, UT, United States) for 48 h using a modified Trowell’s culture method as previously described (Kim et al., 2009). The cultured tooth germs were then transplanted into the kidney capsule of 7 week old male mice for one and 3 weeks to obtain the calcified teeth as described previously (Neupane et al., 2020). The harvested renal calcified teeth were photographed using a Leica MZ16FA.

### Histology and immunohistochemistry

The *in vitro* cultivated tooth germs and renal calcified teeth were sectioned for histology and immunohistochemical analysis. Before sectioning, the one and 3 week calcified teeth were decalcified using 0.5 M EDTA. The histology and immunohistochemistry (IHC) were performed as described previously (Neupane et al., 2020). For histological analysis, Hematoxylin and eosin (H&E) staining and Masson’s trichrome (MTC) staining were performed. Primary antibodies used in this study are Nestin (Abcam, Cambridge, United Kingdom, 1:400; cat. no. ab11306), Amelogenin (AMELX; Abcam, Cambridge, United Kingdom, 1:500; cat. no. ab153915), Glucose regulatory protein 78 (GRP78; Abcam, Cambridge, United Kingdom, 1:400; cat. no. ab21685) and HRD1 (Novus Biologicals, Colorado, United States, 1:200; cat. no. NB100-2526) and Ki67 (Thermo Scientific, Waltham, MA, United States, 1:400; cat. no. RM-9106-s). 1X western blocking solution (Roche, Mannheim, Germany; Ref. 11921673001) was treated for blocking of non-specific binding of antibody. Similarly, the biotinylated anti-rabbit or anti-rat IgG were used as secondary antibodies. The antibody binding was visualized using the diaminobenzidine tetrahydrochloride reagent kit (GBI Labs, Bothell, WA, United States, cat no. C09-12). The images were photographed using DM2500 microscope (Leica, Wetzlar, Germany).

### Quantitative PCR (qPCR)

RNA extraction from *in vitro* cultivated tooth germs and cDNA synthesis for qPCR analysis were performed as described previously (Aryal et al., 2020). The data of real time PCR were normalized to hypoxanthine-guanine phosphoribosyltransferase (Hprt) gene and are expressed as mean ± standard deviation. The primers used during qPCR experiment are presented inSupplementary Table S1.

### TUNEL assay

TUNEL assay was performed using *in situ* cell apoptosis detection kit (Trevigen, Gaithersburg, MD, United States, cat. no. 4810-30-K) as described previously (Neupane et al., 2014). The images were photographed using DM2500 microscope (Leica, Wetzlar, Germany).

### *In situ* hybridization

To examine the expression patterns of genes, whole mount and section *in situ* hybridizations were performed in the *in vitro* cultivated tooth germs as described previously (Sohn et al., 2012). For hybridization, digoxigenin-labeled mRNA probes were hybridized overnight at 65°C. The images were photographed using MZ16FA and DM2500 microscope (Leica, Wetzlar, Germany).

### Three-dimensional (3D) reconstruction

For 3D reconstruction, the serial sections of cultivated tooth with thickness of 7 μm were photographed. The tooth germs were then reconstructed for each specimen using “Voloom 2.3” software (Micro Dimensions, Germany). The images were then aligned automatically and manually.

### Statistical analysis

ImageJ software (http://imagej.net/) was used to count the immunostaining and TUNEL positive cells as described previously (Neupane et al., 2020). The number of Ki67 and TUNEL positive cells in the DAB-stained sections were counted in the defined area of 50 × 50 μm^2^. The number of Ki67 positive cells were counted from the epithelium and adjacent mesenchyme, whereas the TUNEL positive cells were counted from the enamel knot area. Similarly, the cusp, crown, root and mesiodistal length of renal calcified teeth were measured using ImageJ (http://imagej.net/) as described previously (Neupane et al., 2020). Data were represented as ± standard deviations and the mean was determined by comparing control and 4PBA treated groups using Student’s t-test. *p* < .05 indicates significance.

## Results

### ER stress in the developing tooth

**FIGURE 1:**
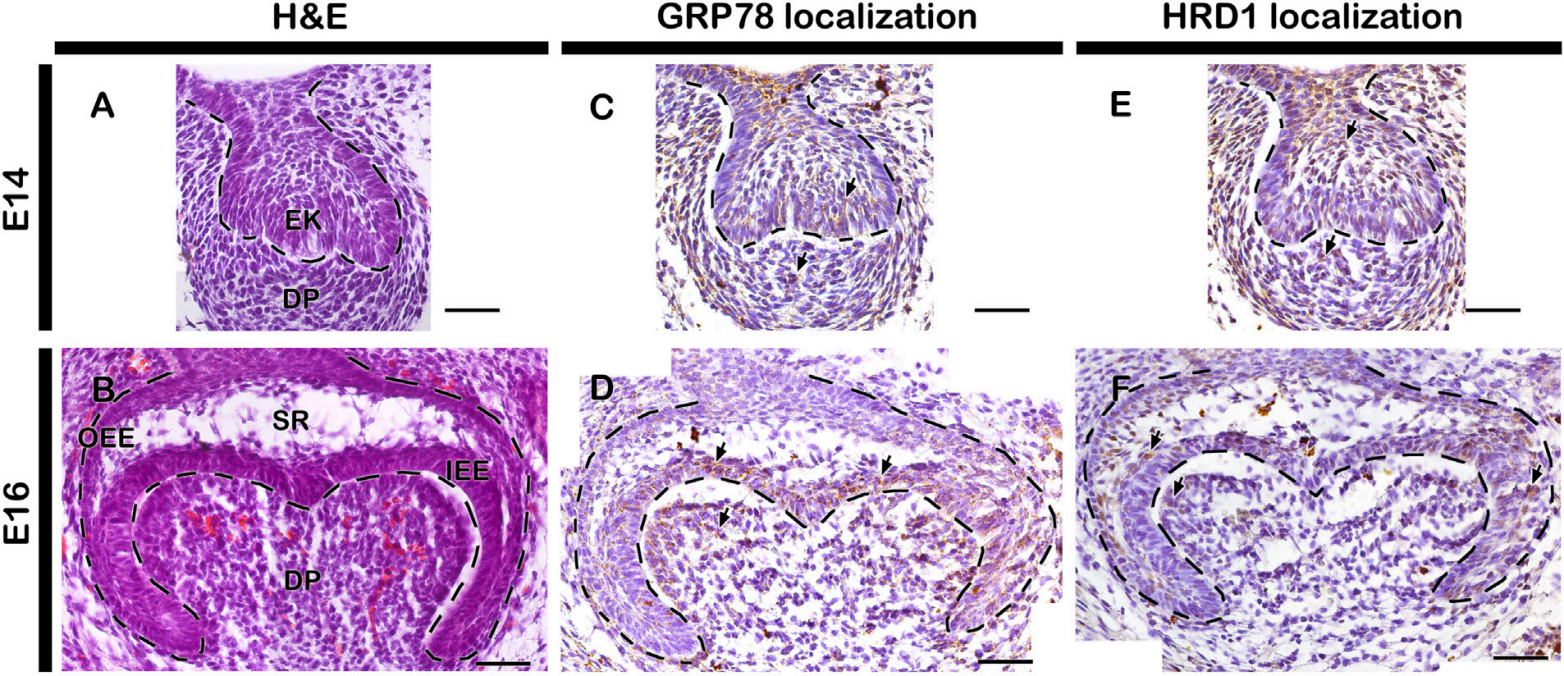
Histology and localization patterns of ER stress markers in mouse developing teeth. HE staining showing embryonic tooth germs at cap **(A)** and bell stage **(B)** of tooth development. Localizations of GRP78 and HRD1 at E14 and E16 tooth germs and counterstained with hematoxylin **(C–F)**. Dotted lines demarcate epithelial boundary **(A–F)**. Arrows indicate immunostaining positive cells. EK, enamel knot; IEE, inner enamel epithelium; OEE, outer enamel epithelium; DP, dental papilla; SR, stellate reticulum. Scale bars: 200 μm **(A,C and E)**, 50 μm **(B,D and F)**.

The normal developing tooth at E14 and E16 represent cap and bell stage respectively (Figures 1A, B). Similar to previous report (Aryal et al., 2 020), the localizations of ER stress related markers: 74-kDa glucose-regulated protein (GRP78) and HMG-CoA reductase degradation protein 1 (HRD1) were observed in the cap and bell stage of tooth development (Figures 1C–F). GRP78, a major ER chaperone protein and a master regulator for ER stress, localized at DP, EK and IEE at cap stage (Figure 1C). However, at bell stage, the localization of GRP78 was restricted only to enamel forming area (Figure 1D). Similarly, HMG-CoA, a key enzyme for ER-associated degradation (ERAD) of misfolded proteins, was localized along the entire tooth germ during cap stage (Figure 1E), whereas, strong localization was observed along the enamel forming area during bell stage (Figure 1F).

### Altered cellular physiology after 4PBA treatment

**FIGURE 2:**
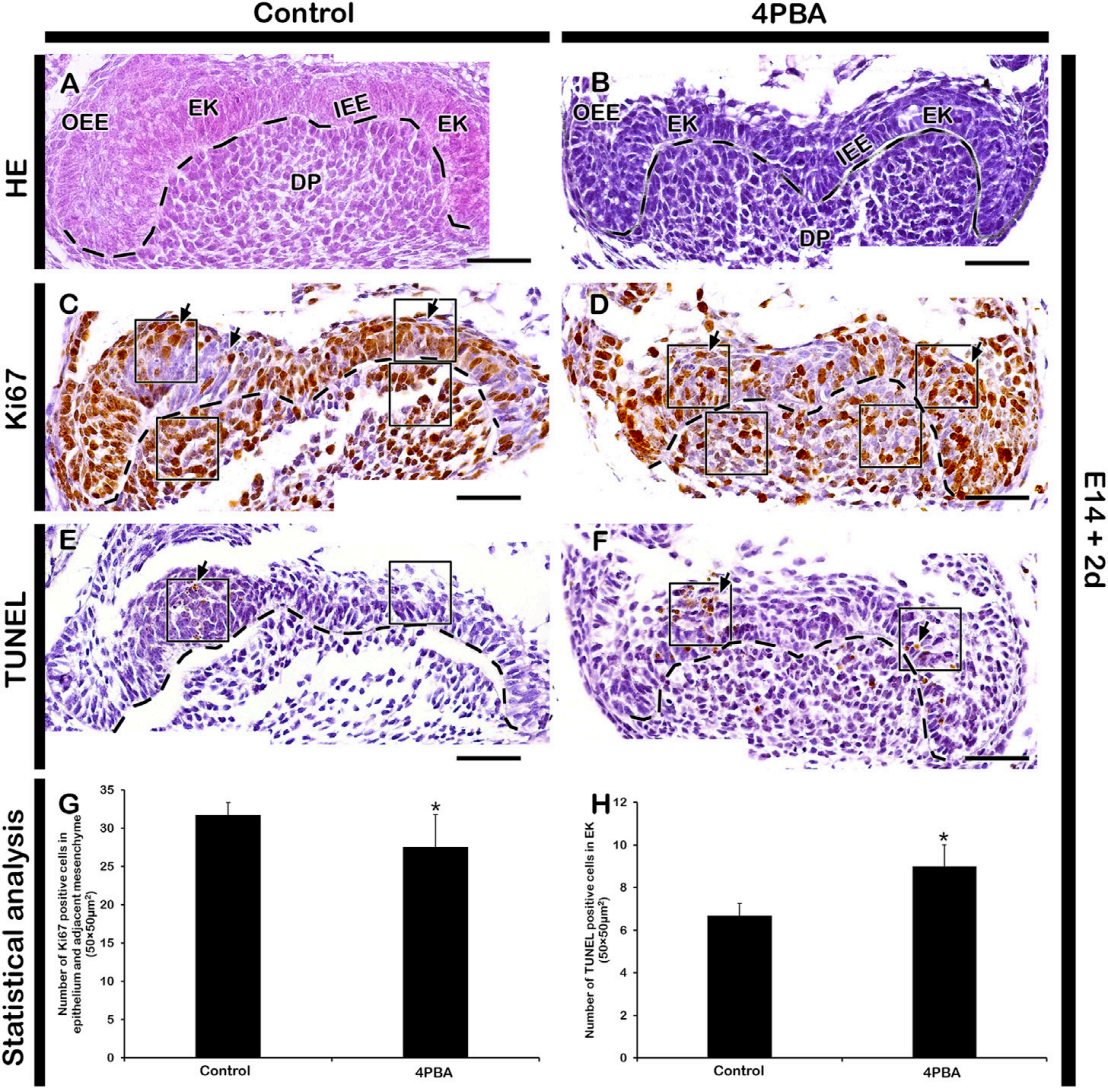
Histology, immunostaining of Ki67 and TUNEL assay of 2 days *in vitro* organ cultivated teeth. HE staining of *in vitro* cultivated teeth **(A,B)**. Ki67 immunostaining **(C,D)** and TUNEL assay **(E,F)** in the control and 4PBA treated group. In the 4PBA treated group, proliferating cells are reduced in the epithelium and adjacent mesenchyme **(C,D)**, however, number of apoptotic cells are increased in epithelium when compared to control (E-F). Tissues are counterstained with hematoxylin **(C–F)**. Statistical analysis showing Ki67 and TUNEL positive cells **(G,H)**. N = 6. * indicates *p* < .05 **(G,H)**. Dotted lines demarcate the epithelial boundary **(A–F)**. Arrows indicate immunostaining and TUNEL positive cells **(E,F)**. Square boxes indicate area of 50 µm^2^ × 50 µm^2^ for positive cell count **(E,F)**. EK, enamel knot; SR, stellate reticulum; IEE, inner enamel epithelium; DP, dental papilla. Scale bars: 50 μm **(A–F)**.

**FIGURE 3:**
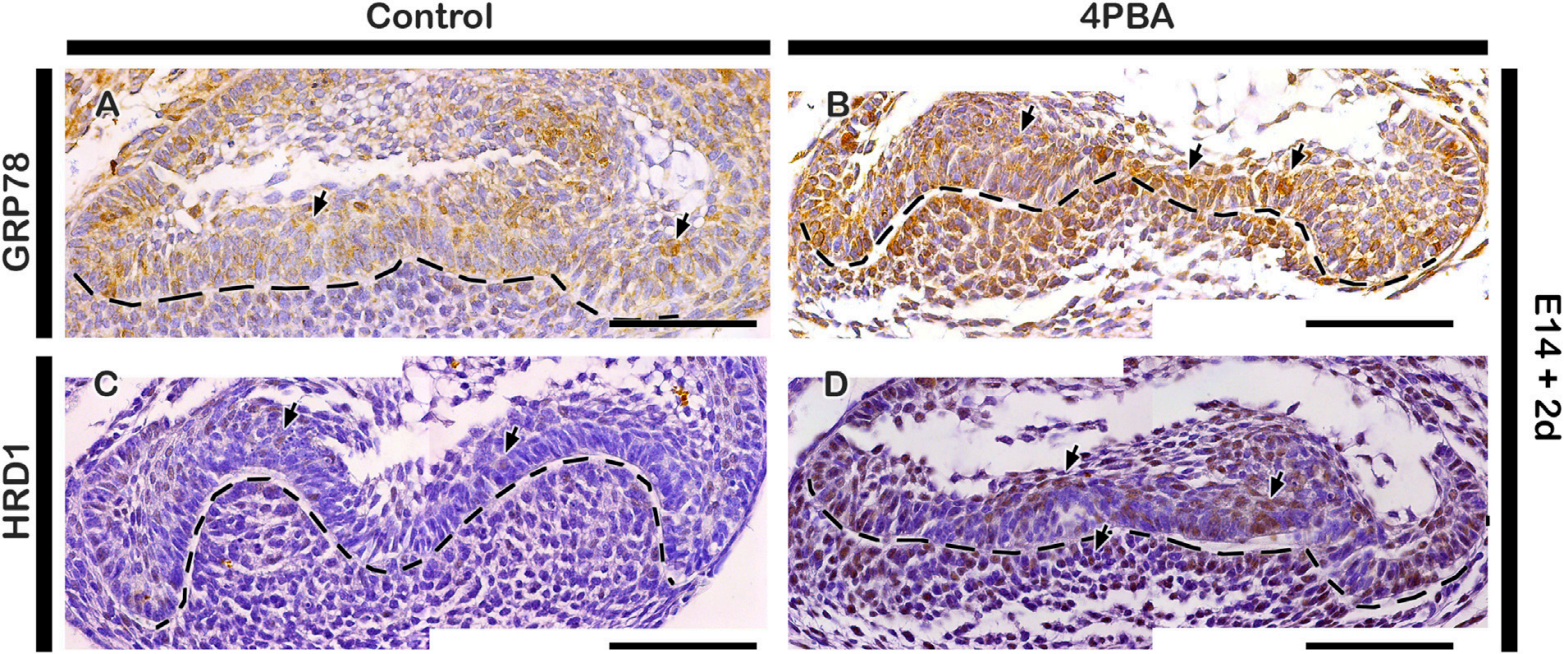
Localization patterns of GRP78 and HRD1. *In vitro* organ cultivation at E14 for 2 days showing increased localizations of GRP78 and HRD1 in both epithelium and mesenchyme of 4PBA treated teeth germs compared to controls **(A–D)**. Tissues are counterstained with hematoxylin **(A–D)**. Arrows indicate immunostaining and TUNEL positive cells **(A–D)**. Dotted lines demarcate the epithelial boundary **(A–D)**. Scale bars: 50 μm **(A–D)**.

To examine the effect of 4PBA in the developing tooth germ, we performed *in vitro* organ cultivation of E14 tooth germs for 2 days. Cellular proliferation were examined by immunostaining against Ki67, and the apoptosis were examined by TUNEL assay (Figures 2A–F). The number of proliferative cells were decreased in the 4PBA treated groups when compared to control (Figures 2C, D, G). Particularly, the EK area showed decreasing number of proliferative cells in the 4PBA treated group (Figures 2A–D). Whereas, apoptotic event of 4PBA treated group showed more apoptotic cells, especially at the EK area when compared to control (Figures 2E, F, H). To compare the exact size of EK, the expression pattern of Fgf4 was examined using whole mount *in situ* hybridization, however, there was no obvious differences between control and 4PBA treated specimens (Supplementary Figure S1), To examine the activity of ER stress during *in vitro* cultivation, we performed immunostainings against GRP78 and HRD1 (Figures 3A–D). The localization of GRP78 in the 4PBA treated group was more intense than those of controls throughout the entire tooth germ (Figures 3 A, B, Supplementary Figure S2). Similarly, the localization of HRD1 was stronger along the IEE and dental papilla in the 4 PBA treated tooth germs when compared to control (Figures 3 C, D, Supplementary Figure S2). We also measured the relative expression levels of signaling molecules related to amelogenesis, odontogenesis and ER stress after 24 h of *in vitro* organ cultivation at E14 with treatment of 4PBA. After 12 h of cultivation, all the molecules were up-regulated with treatment of 4PBA, however, their expressions levels did not altered significantly after 24 h (data not shown).

### Tooth phenotype after 4PBA treatment

**FIGURE 4:**
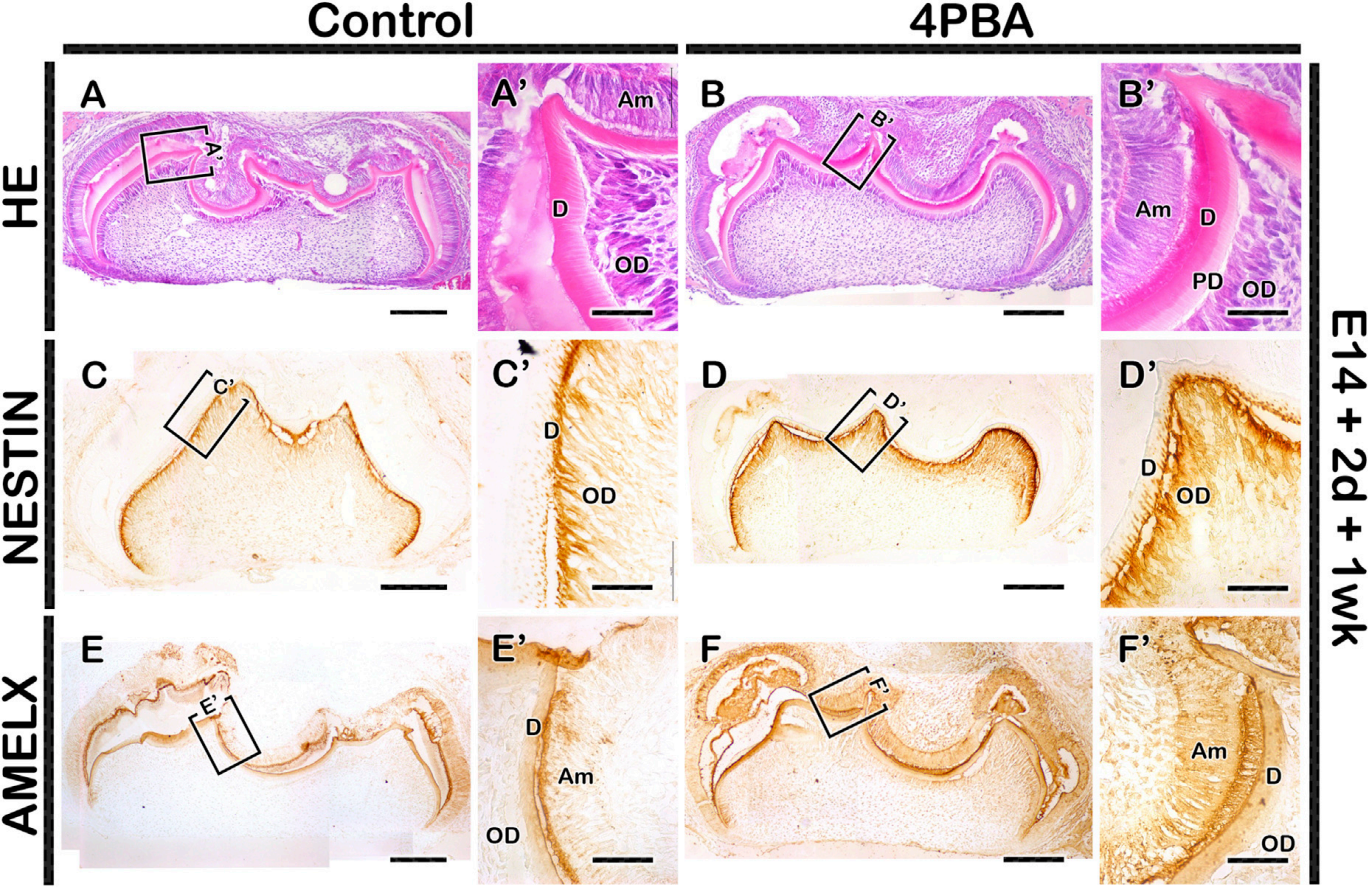
Histology and localization patterns of NESTIN and AMELX in renal calcified teeth. HE staining showing comparably thicker enamel and dentin layers in the 4PBA treated teeth compared to controls **(AA′,BB′)**. Similarly, the localization patterns of NESTIN and AMELX are stronger in the 4PBA treated teeth when compared to control group **(CC′–FF′)**. Boxes indicate enlarged views **(A–F)**. Am, ameloblast; OD, odontoblast; PD, predentin; D, dentin. Scale bars: 200 μm **(A–F)**, 50 μm **(A′–F′)**.

**FIGURE 5:**
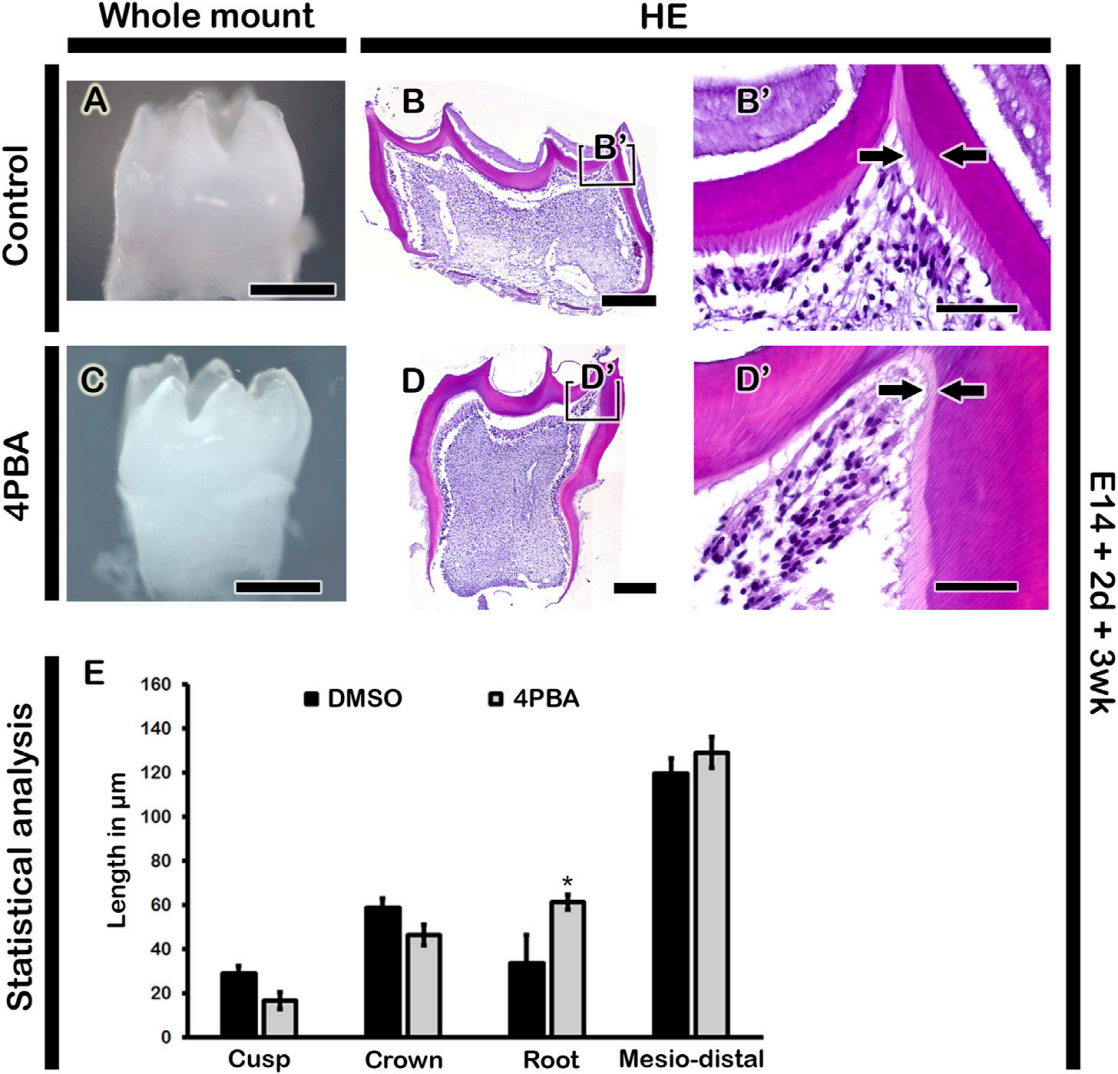
Morphology and histology of renal calcified teeth. The 3-week renal calcified teeth showing elongated tooth roots in the 4PBA treated group when compared to control **(A, C)**. Thicker predentin in the cuspal area in the 4PBA treated teeth **(B′, D′)**. Boxes in B and D indicate enlarged views. Arrows indicate thickness of predentin **(B′, D′)**. Scale bars: 500 μm **(A, C)**, 200 μm **(B, D)**, 50 μm **(B′, D′)**. Statistical analysis showing cusp, crown, root and mesio-distal lengths in the control and 4PBA treated group **(E)**. N = 5. * indicates *p* < 0.05.

To examine the long term effect of 4PBA in tooth phenotype, we performed kidney capsule transplantation for one and 3 week after *in vitro* cultivation of tooth germs at E14 for 2 days. One week renal calcified teeth displayed denser alignment of odontoblasts with thicker dentin and enamel space in the 4PBA treated teeth when compared to control (Figures 4A, A′, B, B′). Meanwhile, the activations of odontoblasts and ameloblasts were examined with immunostainings against NESTIN and AMELOGENIN (Figures 4C, C′–F, F′). 4PBA treated group showed apparently stronger localization pattern of NESTIN in active odontoblasts when compared to control group (Figures 4C, C′, D, D′). Similarly, strong localization pattern of AMELOGENIN in the 4PBA treated group was observed (Figures 4E, E′, F, F′). Moreover, 3 weeks renal calcified teeth showed altered morphological changes in the 4PBA treated teeth (Figures 5A–D). Particularly, thicker predentin was observed along the cuspal area in the 4PBA treated teeth when compared to control (Figures 5A–D). To compare the control and 4PBA treated teeth precisely, we randomly selected 5 teeth from both groups and measured the cusp, crown, root and mesio-distal length. Interestingly, 4PBA treated group showed shorter cusp and crown length, however, the root and mesio-distal lengths were longer when compared to control (Figure 5E). Especially, root length of 4PBA treated tooth was almost double than that of control group (Figure 5E).

## Discussion

ER stress pathway is involved during development and differentiation of hard tissue formation (Cameron et al., 2015; Li et al., 2018; Chen et al., 2020). Especially in tooth development, ER stress plays a key role in the ameloblast (Brookes et al., 2014; Suzuki et al., 2017; Aryal et al., 2019, 2020) and odontoblast (Kim Y.-G. et al., 2014) differentiation. In addition, ER stress molecules play a vital role in maintaining cellular homeostasis during odontogenesis because their expression patterns coincide with the novel transcription-regulating signaling molecules (Uchibe et al., 2012; Aryal et al., 2020). Particularly, the role of UPR in cap stage is not only alleviating protein overload in the ER, but also interacting with signaling molecules for cellular homeostasis during cap to bell stage transition (Aryal et al., 2020). In addition, the expressions of ER stress regulator genes: Ire1 and Xbp1 at IEE and dental papilla during bell stage suggest that Ire1/Xbp1 ER stress pathway initiates the ameloblast precursor cells to secrete ECM and dental papilla cells to differentiate into odontoblasts beforehand (Tsuchiya et al., 2008; Brookes et al., 2014; Kawashima and Okiji, 2016; Aryal et al., 2020), thereby emphasizing the importance of ER stress molecules and chemical chaperones during extracellular matrix secretion and tooth morphogenesis. Therefore, this study examined the effects of chemical chaperone, 4PBA, during tooth development using *in vitro* organ cultivation system.

The clinical trials of 4PBA in both *in vitro* and *in vivo* as one of the potential therapeutic agent for metabolic diseases such as obesity and diabetes, inflammatory disorders and cancers has been studied (Luo et al., 2010; Iannitti and Palmieri, 2011; Kim et al., 2013; Chen et al., 2016). Especially, 4PBA rescued malformed tooth enamel, mirroring human AI and improved the gross appearance of incisor teeth of (Barron et al., 2010; Brookes et al., 2014). According to Brookes et al., 4PBA does not directly interact with secretory load of ER to restore normal secretory function, but it appears to inhibit ameloblast apoptosis itself (Brookes et al., 2014). In our study, after treatment of 4PBA, localization of ER stress markers: GRP78 and HRD1, as well as NESTIN and AMELOGENIN were increased with denser alignment of odontoblasts and thicker dentin/enamel space (Figures 3, 4). These results suggest that chemical chaperone (4PBA) facilitates matrix formation in normal tooth development, and as a result of extensive secretion of matrices, ER stress was increased than in normal developing condition. Moreover, alteration in tooth phenotype was due to alteration in cellular events such as proliferation, apoptosis and ER stress in the 4PBA treated teeth (Figures 2, 3) because the alteration in enamel knot signaling play a vital role for future cusp, crown and root morphogenesis (Cho et al., 2007; Aryal et al., 2020; Neupane et al., 2020). The longer root length and thicker matrices of predentin in the 4PBA treated specimen suggest that this chemical chaperone facilitates the transcription of genes during development of tooth root and secretion of matrices because 4PBA as a histone deacetylase inhibitor can possibly facilitate transcription of multiple genes which regulate apoptosis and promote survival in the formation of matrices (Duncan et al., 2013; Brookes et al., 2014; Zhang and Zhong, 2014; Chotikatum et al., 2018).

The application of 4PBA as a drug repositioning method in dental procedures has been practiced for dentin regeneration (Lee et al., 2022). Based on these results, we speculated that proper amount of concentrated drug, 4PBA would affect tooth development in fetal period. The *in vitro* organ cultivation using embryonic stage tooth may imitate human oral environment of tooth transition period because permanent teeth in human initiate to develop in the jaws at birth and continue after a child is born. Our results suggest that direct absorption of chemical chaperone in oral environment during normal primary tooth development may affect matrix formation of enamel and dentin. Additionally, we speculated that disorders like dentin dysplasia type I which is characterized by short or absence of tooth roots, obliterated pulp chambers (Malik et al., 2015) would be improved through exposure of chemical chaperone, such as 4PBA during permanent tooth development period.

In summary, ER stress occurs inevitably during secretory stage of tooth development. When protein folding capacity is overloaded, the UPR is activated which functions as a significant factor for normal tooth development. Our results showed the alterations in cellular physiology and morphology of developing tooth when treated with chemical chaperone, 4PBA. These results imply that when 4PBA is treated during tooth development period, or specifically during human tooth transition period, there would be both positive and negative effects of chemical chaperone. Further studies about these effects need to be proceeded in the near future.

## Data Availability

The original contributions presented in the study are included in the article/Supplementary Material, further inquiries can be directed to the corresponding authors.

## References

[B1] Abramyan J. Geetha-Loganathan P. Šulcová M. Buchtová M. (2021). Role of cell death in cellular processes during odontogenesis. Front. Cell Dev. Biol. 9, 671475. 10.3389/fcell.2021.671475 34222243 PMC8250436

[B2] Aryal Y. P. Lee E.-S. Kim T.-Y. Sung S. Kim J.-Y. An S.-Y. (2020). Stage-specific expression patterns of ER stress-related molecules in mice molars: Implications for tooth development. Gene Expr. Patterns 37, 119130. 10.1016/j.gep.2020.119130 32758541

[B3] Aryal Y. P. Neupane S. Adhikari N. An C.-H. Ha J.-H. Kwon T.-Y. (2019). An endoplasmic reticulum stress regulator, Tmbim6, modulates secretory stage of mice molar. J. Cell. Physiol. 234, 20354–20365. 10.1002/jcp.28635 30963569

[B4] Balic A. (2018). Biology explaining tooth repair and regeneration: A mini-review. Gerontology 64, 382–388. 10.1159/000486592 29533942

[B5] Barron M. J. Brookes S. J. Kirkham J. Shore R. C. Hunt C. Mironov A. (2010). A mutation in the mouse Amelx tri-tyrosyl domain results in impaired secretion of amelogenin and phenocopies human X-linked amelogenesis imperfecta. Hum. Mol. Genet. 19, 1230–1247. 10.1093/hmg/ddq001 20067920 PMC2838535

[B6] Brookes S. J. Barron M. J. Boot-Handford R. Kirkham J. Dixon M. J. (2014). Endoplasmic reticulum stress in amelogenesis imperfecta and phenotypic rescue using 4-phenylbutyrate. Hum. Mol. Genet. 23, 2468–2480. 10.1093/hmg/ddt642 24362885 PMC3976337

[B7] Brookes S. J. Barron M. J. Dixon M. J. Kirkham J. (2017). The unfolded protein response in amelogenesis and enamel pathologies. Front. Physiol. 8, 653. 10.3389/fphys.2017.00653 28951722 PMC5599773

[B8] Cameron T. L. Gresshoff I. L. Bell K. M. Piróg K. A. Sampurno L. Hartley C. L. (2015). Cartilage-specific ablation of XBP1 signaling in mouse results in a chondrodysplasia characterized by reduced chondrocyte proliferation and delayed cartilage maturation and mineralization. Osteoarthr. Cartil. 23, 661–670. 10.1016/j.joca.2015.01.001 25600960

[B9] Chen Y. Guo Y. Li J. Chen Y.-Y. Liu Q. Tan L. (2020). Endoplasmic reticulum stress remodels alveolar bone formation after tooth extraction. J. Cell. Mol. Med. 24, 12411–12420. 10.1111/jcmm.15753 32996245 PMC7687007

[B10] Chen Y. Wu Z. Zhao S. Xiang R. (2016). Chemical chaperones reduce ER stress and adipose tissue inflammation in high fat diet-induced mouse model of obesity. Sci. Rep. 6, 27486. 10.1038/srep27486 27271106 PMC4897685

[B11] Cho S. W. Lee H. A. Cai J. Lee M. J. Kim J. Y. Ohshima H. (2007). The primary enamel knot determines the position of the first buccal cusp in developing mice molars. Differentiation 75 (5), 441–451. 10.1111/j.1432-0436.2006.00153.x 17309607

[B12] Chotikatum S. Naim H. Y. El-Najjar N. (2018). Inflammation induced ER stress affects absorptive intestinal epithelial cells function and integrity. Int. Immunopharmacol. 55, 336–344. 10.1016/j.intimp.2017.12.016 29324356

[B13] Cortez L. Sim V. (2014). The therapeutic potential of chemical chaperones in protein folding diseases. Prion 8, 197–202. 10.4161/pri.28938 24818993 PMC4189890

[B14] Duncan H. F. Smith A. J. Fleming G. J. P. Cooper P. R. (2013). Histone deacetylase inhibitors epigenetically promote reparative events in primary dental pulp cells. Exp. Cell Res. 319, 1534–1543. 10.1016/j.yexcr.2013.02.022 23562654

[B19] Iannitti T. Palmieri B. (2011). Clinical and experimental applications of sodium phenylbutyrate. Drugs R. D. 11, 227–249. 10.2165/11591280-000000000-00000 21902286 PMC3586072

[B21] Jussila M. Thesleff I. (2012). Signaling networks regulating tooth organogenesis and regeneration, and the specification of dental mesenchymal and epithelial cell lineages. Cold Spring Harb. Perspect. Biol. 4, a008425. 10.1101/cshperspect.a008425 22415375 PMC3312678

[B22] Kawashima N. Okiji T. (2016). Odontoblasts: Specialized hard-tissue-forming cells in the dentin-pulp complex. Congenit. Anom. (Kyoto). 56, 144–153. 10.1111/cga.12169 27131345

[B23] Kim H. J. Jeong J. S. Kim S. R. Park S. Y. Chae H. J. Lee Y. C. (2013). Inhibition of endoplasmic reticulum stress alleviates lipopolysaccharide-induced lung inflammation through modulation of NF-κB/HIF-1α signaling pathway. Sci. Rep. 3, 1142. 10.1038/srep01142 23359618 PMC3556596

[B24] Kim J.-Y. Lee M.-J. Cho K.-W. Lee J.-M. Kim Y.-J. Kim J.-Y. (2009). Shh and ROCK1 modulate the dynamic epithelial morphogenesis in circumvallate papilla development. Dev. Biol. 325, 273–280. 10.1016/j.ydbio.2008.10.034 19014928

[B25] Kim J. W. Choi H. Jeong B. C. Oh S. H. Hur S. W. Lee B. N. (2014a). Transcriptional factor ATF6 is involved in odontoblastic differentiation. J. Dent. Res. 93, 483–489. 10.1177/0022034514525199 24570149 PMC6728569

[B26] Kim Y.-G. Park J.-W. Lee J.-M. Suh J.-Y. Lee J.-K. Chang B.-S. (2014b). IL-17 inhibits osteoblast differentiation and bone regeneration in rat. Arch. Oral Biol. 59, 897–905. 10.1016/j.archoralbio.2014.05.009 24907519

[B27] Li H. Li D. Ma Z. Qian Z. Kang X. Jin X. (2018). Defective autophagy in osteoblasts induces endoplasmic reticulum stress and causes remarkable bone loss. Autophagy 14, 1726–1741. 10.1080/15548627.2018.1483807 29962255 PMC6135623

[B28] Luo Z.-F. Feng B. Mu J. Qi W. Zeng W. Guo Y.-H. (2010). Effects of 4-phenylbutyric acid on the process and development of diabetic nephropathy induced in rats by streptozotocin: Regulation of endoplasmic reticulum stress-oxidative activation. Toxicol. Appl. Pharmacol. 246, 49–57. 10.1016/j.taap.2010.04.005 20399799

[B29] Malik S. Gupta S. Wadhwan V. Suhasini G. P. (2015). Dentin dysplasia type I–A rare entity. J. Oral Max Path JOMFP. 19, 110. 10.4103/0973-029X.157220 PMC4451656 26097326

[B30] Neupane S. Aryal Y. P. Kim T.-Y. Yeon C.-Y. An C.-H. Kim J.-Y. (2020). Signaling modulations of miR-206-3p in tooth morphogenesis. Int. J. Mol. Sci. 21, 5251. 10.3390/ijms21155251 32722078 PMC7432545

[B31] Neupane S. Sohn W.-J. Rijal G. Lee Y.-J. Lee S. Yamamoto H. (2014). Developmental regulations of Perp in mice molar morphogenesis. Cell Tissue Res. 358, 109–121. 10.1007/s00441-014-1908-7 24865245

[B32] Oakes S. A. Papa F. R. (2015). The role of endoplasmic reticulum stress in human pathology. Annu. Rev. Pathol. 10, 173–194. 10.1146/annurev-pathol-012513-104649 25387057 PMC5568783

[B33] Otsu K. Kishigami R. Fujiwara N. Ishizeki K. Harada H. (2011). Functional role of Rho-kinase in ameloblast differentiation. J. Cell. Physiol. 226, 2527–2534. 10.1002/jcp.22597 21792909

[B34] Pobre K. F. R. Poet G. J. Hendershot L. M. (2019). The endoplasmic reticulum (ER) chaperone BiP is a master regulator of ER functions: Getting by with a little help from ERdj friends. J. Biol. Chem. 294, 2098–2108. 10.1074/jbc.REV118.002804 30563838 PMC6369273

[B37] Sano R. Reed J. C. (2013). ER stress-induced cell death mechanisms. Biochim. Biophys. Acta - Mol. Cell Res. 1833, 3460–3470. 10.1016/j.bbamcr.2013.06.028 PMC3834229 23850759

[B38] Santosh A. B. R. Jones T. J. (2014). The epithelial-mesenchymal interactions: Insights into physiological and pathological aspects of oral tissues. Oncol. Rev. 8, 239. 10.4081/oncol.2014.239 25992230 PMC4419607

[B39] Smith C. E. L. Poulter J. A. Antanaviciute A. Kirkham J. Brookes S. J. Inglehearn C. F. (2017). Amelogenesis imperfecta; genes, proteins, and pathways. Front. Physiol. 8, 435. 10.3389/fphys.2017.00435 28694781 PMC5483479

[B40] Sohn W.-J. Ji Y.-R. Kim H.-S. Gwon G.-J. Chae Y.-M. An C.-H. (2012). Rgs19 regulates mouse palatal fusion by modulating cell proliferation and apoptosis in the MEE. Mech. Dev. 129, 244–254. 10.1016/j.mod.2012.07.004 22841956

[B41] Suzuki M. Everett E. T. Whitford G. M. Bartlett J. D. (2017). 4-phenylbutyrate mitigates fluoride-induced cytotoxicity in ALC cells. Front. Physiol. 8, 302. 10.3389/fphys.2017.00302 28553235 PMC5425599

[B43] Tabas I. Ron D. (2011). Integrating the mechanisms of apoptosis induced by endoplasmic reticulum stress. Nat. Cell Biol. 13, 184–190. 10.1038/ncb0311-184 21364565 PMC3107571

[B44] Tsuchiya M. Tye C. E. Sharma R. Smith C. E. Bartlett J. D. (2008). XBP1 may determine the size of the ameloblast endoplasmic reticulum. J. Dent. Res. 87, 1058–1062. 10.1177/154405910808701115 18946015 PMC2593005

[B45] Uchibe K. Shimizu H. Yokoyama S. Kuboki T. Asahara H. (2012). Identification of novel transcription-regulating genes expressed during murine molar development. Dev. Dyn. 241, 1217–1226. 10.1002/dvdy.23808 22639370

[B46] Uppala J. K. Gani A. R. Ramaiah K. V. A. (2017). Chemical chaperone, TUDCA unlike PBA, mitigates protein aggregation efficiently and resists ER and non-ER stress induced HepG2 cell death. Sci. Rep. 7, 3831. 10.1038/s41598-017-03940-1 28630443 PMC5476595

[B47] Wu T. Zhang S. Xu J. Zhang Y. Sun T. Shao Y. (2020). HRD1, an important player in pancreatic β-cell failure and therapeutic target for type 2 diabetic mice. Diabetes 69, 940–953. 10.2337/db19-1060 32086291

[B48] Yu T. Klein O. (2020). Molecular and cellular mechanisms of tooth development, homeostasis and repair. Development 147, dev184754. 10.1242/dev.184754 31980484 PMC6983727

[B49] Zhang H. M. Ye X. Su Y. Yuan J. Liu Z. Stein D. A. (2010). Coxsackievirus B3 infection activates the unfolded protein response and induces apoptosis through downregulation of p58IPK and activation of CHOP and SREBP1. J. Virol. 84, 8446–8459. 10.1128/JVI.01416-09 20554776 PMC2918999

[B50] Zhang J. Zhong Q. (2014). Histone deacetylase inhibitors and cell death. Cell. Mol. Life Sci. 71, 3885–3901. 10.1007/s00018-014-1656-6 24898083 PMC4414051

