# Supplementary material for: The effects of 4-Phenylbutyric acid on ER stress during mouse tooth development
Source: Front Physiol. 2023 Jan 4;13:1079355. doi: 10.3389/fphys.2022.1079355 (PMC9848431; doi:10.3389/fphys.2022.1079355)
Supplement: Supplementary file 1 [file Table1.DOCX]

**Supplementary figures**


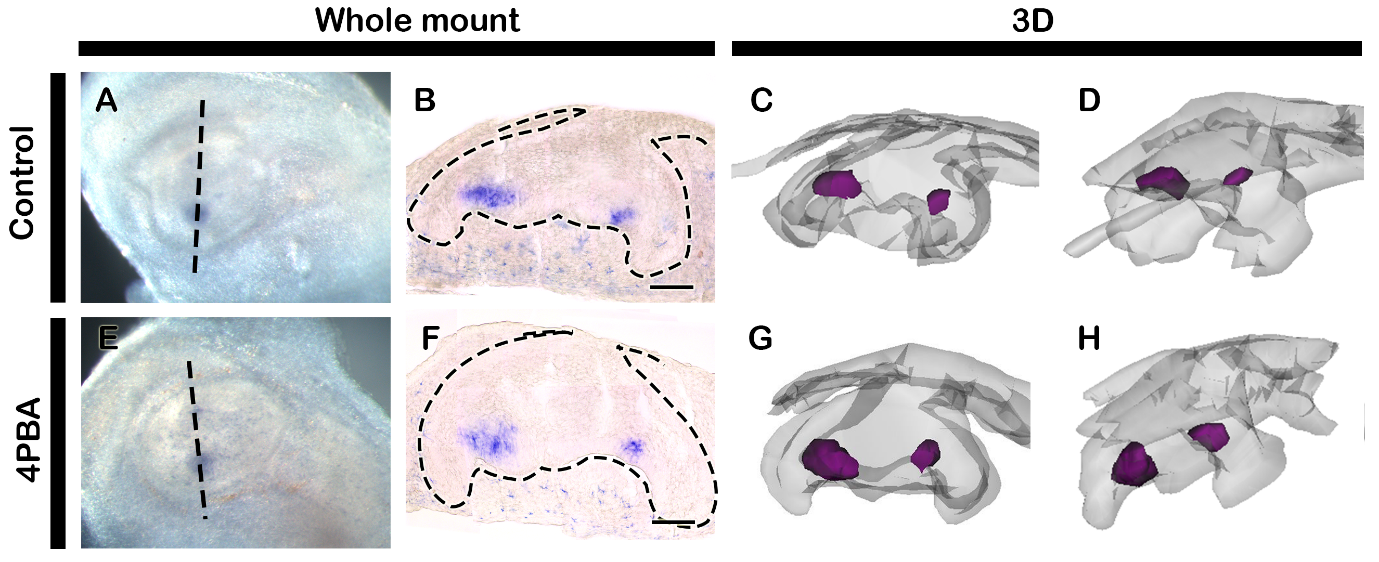


Figure S1. Whole mount in situ hybridization of Fgf4 in the 2-day in vitro organ cultivated tooth germs (A-B, E-F). The expression of Fgf4 is almost similar in both group. 3D- reconstruction showing similar size of enamel knots in control and 4PBA group (C-D, G-H). Dotted lines indicate section views (A, E) and epithelial boundary (B, F). Scale bars: 50 μm (B, F).

**Table S1. List of primers used in the study**

| **Gene** | Accession | Primer sequence | Product size |
| --- | --- | --- | --- |
| **Ambn** | NM_001303431.1 | Forward: TTCTTGCTTTCCCCAATGAC | 234 |
|  |  | Reverse: GGTGCACTTTGTTTCCAGGT |  |
| **Amelx** | XM_017348358,1 | Forward: GCAGCCGTATCCTTCCTATGGTT | 120 |
|  |  | Reverse: GGAAGGTGGTGATGAGGCTGAA |  |
| **Atf6** | NM_001081304.1 | Forward: GCAGATTGACTGTCAGGTGA | 206 |
|  |  | Reverse: TCTTCAGTCTCTCGTGTCCA |  |
| **Bmp2** | NM_007553.3 | Forward: AAGTGGCCCATTTAGAGGAG | 104 |
|  |  | Reverse: CAATGGCCTTATCTGTGACC |  |
| **Bmp4** | NM_007554.2 | Forward: ACCTCAAGGGAGTGGAGATT | 113 |
|  |  | Reverse: GATGCTTGGGACTACGTTTG |  |
| **Dmp1** | NM_001359013.1 | Forward: CAGAGGGACAGGCAAATAGTGAC | 168 |
|  |  | Reverse: CATCGCCAAAGGTATCATCTCC |  |
| **Dspp** | NM_010080.3 | Forward: GGCTCCGAGTCAATACATGTA Reverse: CTCCTTGGTGTCCATTGCTAT | 933 |
| **Enam** | NM_017468.3 | Forward: GCCCCACCAATGATGCCTAT | 200 |
|  |  | Reverse: TGTGGATTGGTCTGGTTGGG |  |
| **Fgf4** | NM_010202.5 | Forward: TCGCCTACCATGAAGGTAAC | 114 |
|  |  | Reverse: TCTCCATCGAGAGAAAGTGC |  |
| **lre1** | NM_012016.3 | Forward: TTTAGCTTTGCCGACCGTGA | 123 |
|  |  | Reverse: TGCACACAGCTCGATAGCAA |  |
| **Perk** | NM_010121.3 | Forward: CACGCAGATCACAGTCAGGT | 166 |
|  |  | Reverse: GGGCTGAGGATGGAAAAGCC |  |
| **Shh** | NM_009170.3 | Forward: CAGCGCGTGTACGTGGTGGC | 335 |
|  |  | Reverse: GGAGCGTCGGCAGCACCTG |  |
| **Hprt** | NM_013556.1 | Forward: CCTAAGATGATCGCAAGTTG | 86 |
|  |  | Reverse: CCACAGGGACTAGAACACCTGCTAA |  |

**Table S2**. Statistical evaluation of immunohistochemical staining against GRP78 and HRD1

| **Group** | **Control** | **4PBA** |
| --- | --- | --- |
| GRP78 | ++ | +++ |
| HRD1 | + | ++ |

-:none, +: exist, ++: strong, +++: strongest
